# Prostate cancer incidence and mortality in men exposed to α1-adrenergic receptor antagonists

**DOI:** 10.1093/jnci/djae108

**Published:** 2024-05-08

**Authors:** Lars Björnebo, Shirin Razdan, Andrea Discacciati, Thorgerdur Palsdottir, Markus Aly, Tobias Nordström, Martin Eklund, Dara Lundon, Henrik Grönberg, Ash Tewari, Peter Wiklund, Natasha Kyprianou, Anna Lantz

**Affiliations:** Department of Medical Epidemiology and Biostatistics, Karolinska Institutet, Stockholm, Sweden; Department of Urology, Icahn School of Medicine at Mount Sinai, New York, NY, USA; Department of Medical Epidemiology and Biostatistics, Karolinska Institutet, Stockholm, Sweden; Department of Medical Epidemiology and Biostatistics, Karolinska Institutet, Stockholm, Sweden; Department of Urology, Karolinska University Hospital Solna, Stockholm, Sweden; Department of Medical Epidemiology and Biostatistics, Karolinska Institutet, Stockholm, Sweden; Department of Medical Epidemiology and Biostatistics, Karolinska Institutet, Stockholm, Sweden; Department of Urology, Icahn School of Medicine at Mount Sinai, New York, NY, USA; Department of Medical Epidemiology and Biostatistics, Karolinska Institutet, Stockholm, Sweden; Department of Urology, Icahn School of Medicine at Mount Sinai, New York, NY, USA; Department of Oncological Science, Icahn School of Medicine at Mount Sinai, New York, NY, USA; Department of Urology, Icahn School of Medicine at Mount Sinai, New York, NY, USA; Department of Urology, Icahn School of Medicine at Mount Sinai, New York, NY, USA; Department of Oncological Science, Icahn School of Medicine at Mount Sinai, New York, NY, USA; Department of Medical Epidemiology and Biostatistics, Karolinska Institutet, Stockholm, Sweden; Department of Urology, Karolinska University Hospital Solna, Stockholm, Sweden

## Abstract

**Background:**

α1-Adrenergic receptor antagonists are commonly used to treat benign prostatic hyperplasia. Preclinical studies suggest that they induce cell death and inhibit tumor growth. This study evaluated the risk of prostate cancer death in men using α1-adrenergic receptor antagonists.

**Methods:**

A population-based cohort study in Stockholm, Sweden (January 1, 2007, to December 31, 2019) included 451 779 men with a prostate-specific antigen test result. Study entry was 1 year after the first prostate-specific antigen test. Men were considered exposed at their second filled prescription. The primary outcome was prostate cancer mortality. Secondary outcomes were all-cause mortality and prostate cancer incidence. Cox proportional hazards regression models were used to calculate adjusted hazard ratios (HRs) and 95% confidence intervals (CIs) for all outcomes. Inverse-probability weighting with marginal structural models accounted for time-dependent confounders.

**Results:**

Of 351 297 men in the final cohort, 39 856 (11.3%) were exposed to α1-adrenergic receptor antagonists. Median (interquartile range) follow-up for prostate cancer mortality was 8.9 (5.1-10.9) years; median (interquartile range) exposure time to α1-adrenergic receptor antagonists was 4.4 (2.0-7.6) years. There was no evidence of an association between α1-adrenergic receptor antagonist use and prostate cancer mortality, all-cause mortality, or high-grade prostate cancer. α1-Adrenergic receptor antagonist use was associated with an increased risk of prostate cancer (HR = 1.11, 95% CI = 1.06 to 1.17) and low-grade prostate cancer (HR = 1.22, 95% CI = 1.11 to 1.33). Men whose prostate cancer was treated with α1-adrenergic receptor antagonists underwent more frequent prostate-specific antigen testing.

**Conclusions:**

Our findings show no significant association between α1-adrenergic receptor adrenoceptor antagonist exposure and prostate cancer mortality or high-grade prostate cancer. Although the preclinical evidence indicates a potential chemopreventive effect, this study’s findings do not support it.

Benign prostatic hyperplasia (BPH) is a common age-related medical condition among men. It affects up to 50% of men older than age 50 years and up to 80% of men older than age 80 years, leading to lower urinary tract symptoms (1,2). BPH prevalence is increasing, largely because of rising modifiable metabolic risk factors such as obesity (3). Medical treatment often involves α1-adrenergic receptor antagonists to relax the smooth muscles at the bladder neck and prostatic urethra by blocking sympathetic activity at these sites (4).

α1-Adrenergic receptor antagonists target 2 types of α1-adrenergic receptor antagonist receptors: α1A and α1B. Adrenoceptors are G-protein–coupled receptors, functionally bound by catecholamines: epinephrine and norepinephrine. The α1A subtype primarily regulates the contraction of smooth muscles in the prostatic urethra and bladder neck; the α1B subtype mainly controls muscle contraction in peripheral blood vessels. Prazosin was the first selective α1A blocker but has largely been replaced by US Food and Drug Administration–approved longer-acting α1-adrenergic receptor antagonists, such as terazosin, doxazosin, tamsulosin, and alfuzosin (4). The selective α1A antagonists have fewer cardiovascular side effects and are preferred for lower urinary tract symptoms associated with BPH (5). Silodosin is a newer selective α1A antagonist with a significantly greater affinity for the α1A receptor than the α1B receptor, making it a promising option with fewer side effects (6).

Prostate cancer is the second-leading cause of cancer-related deaths among men in the United States (7). Current guidelines from the American Urological Association strongly recommend shared decision making for men aged 55 to 69 years considering prostate-specific antigen (PSA) screening, weighing the benefits of reducing the rate of metastatic prostate cancer and prevention of prostate cancer death against the known potential harms associated with screening and treatment (8). There are currently no Food and Drug Administration–approved or European Medicines Agency–approved medications for prostate cancer chemoprevention.

Emerging data from cellular and preclinical models indicate that α1-adrenergic receptor antagonists have additional effects related to inducing apoptosis and suppressing prostate tumor growth and metastasis (9). Epidemiological evidence has thus far been inconclusive regarding the incidence and mortality of prostate cancer among α1-adrenergic receptor antagonist users (10-14).

More than 2 decades have passed since the initial discovery of the molecular and cell-level effects of α1-adrenergic receptor antagonists on prostate tumor growth; however, the impact of these medications on clinical prostate cancer incidence and progression in men remains largely undefined and poorly understood. Considering the conflicting evidence, the primary aim of this large, population-based cohort study was to investigate the possible relationship between the use of α1-adrenergic receptor antagonists and prostate cancer mortality (PCM) as well as its impact on prostate cancer incidence and all-cause mortality.

## Methods

### Data sources study cohort

The Stockholm PSA and Biopsy Register is a population-based register, with near-complete coverage of PSA tests, prostate biopsies, and family history of prostate cancer in Stockholm County since 2003; this register was the source of the study cohort (15). Exposure to α1-adrenergic receptor antagonists and 5-α reductase inhibitors was extracted from the Swedish Prescribed Drug Register, which includes all prescriptions filled at Swedish pharmacies since July 2005 (16). Diagnosed prostate cancers were obtained from the National Prostate Cancer Register, which contains Gleason score and TNM stage for all diagnosed prostate cancers in Sweden (17). The primary cause of death was sourced from the Swedish Cause of Death Register. The Swedish National Patient Register was used to obtain other relevant medical diagnoses (18). Socioeconomic factors (level of education and civil status) were found in the Longitudinal Integrated Database for Health Insurance and Labour Market Studies (19). The regional ethics board in Stockholm, Sweden, approved this study, and informed consent was waived because the analyzed data were deidentified (institutional review board No. 2012/438-31/3).

**Figure 1.**
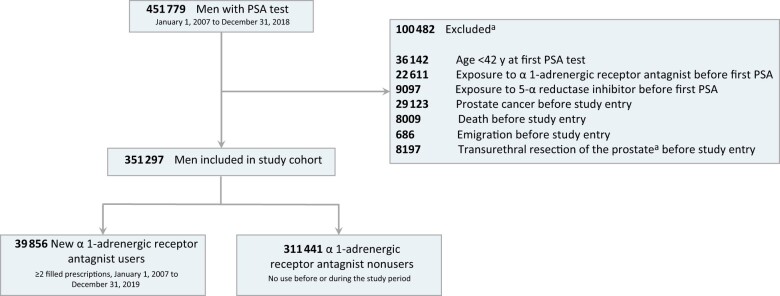
Flowchart for the inclusion and exclusion of patients in the study cohort. ^a^ Groups are not mutually exclusive. PSA = prostate-specific antigen.

For the analysis, men aged 40 years and older with their initial recorded PSA test result between January 2007 and December 2017 were selected. Follow-up started 1 year after the PSA test to exclude men immediately diagnosed with prostate cancer. This scenario could occur when a man is prescribed an α1-adrenergic receptor antagonist or a 5-α reductase inhibitor and receives a prostate cancer diagnosis as part of the initial clinical evaluation of his symptoms. Exclusion criteria included exposure to α1-adrenergic receptor antagonist or 5-α reductase inhibitor before the first PSA test, death or emigration, transurethral resection of the prostate, or prostate cancer diagnosis before study entry (Figure 1). The follow-up period ended at the time of death, emigration, or the conclusion of the study on December 31, 2019.

### Exposure measurement and definition

At the initial PSA test, all patients were categorized as nonusers of both α1-adrenergic receptor antagonists and 5-α reductase inhibitors, with a minimum washout period of 1.5 years (the time difference between the initiation of the Swedish Prescribed Drug Register in July 2005 and the first possible PSA test in January 2007). Subsequently, patients could be classified as users or nonusers of both medications. A nonuser was reclassified as a user once 2 prescriptions had been filled on separate occasions at any time after the initial PSA test. Once a nonuser became a user, the status remained unchanged. Men with only 1 filled prescription remained nonusers. The considered α1-adrenergic receptor antagonists included alfuzosin, terazosin, and tamsulosin; the considered 5-α reductase inhibitors were finasteride and dutasteride.

### Baseline covariates and time-dependent covariates

Many covariates were considered potential confounders for the association between α1-adrenergic receptor antagonists and the outcomes. These covariates were divided into baseline covariates and time-dependent covariates. Baseline covariates were assessed at the time of the first recorded PSA test and included age at the initial PSA screening, PSA level, previous negative biopsy (yes or no), prostate cancer in first-degree relatives (yes or no), the highest level of education obtained (elementary school, high school, university or college), civil status (single or partnered), Charlson Comorbidity Index (0, 1, or ≥2), and year of study entry (2008-2011, 2012-2015, 2016-2019). Time-dependent covariates included exposure to α1B receptors and 5-α reductase inhibitors, PSA levels, and the cumulative number of PSA tests and were updated each time a change in the covariate was observed.

### Statistical analyses

To investigate the association between α1-adrenergic receptor use and prostate cancer detection, all-cause mortality, and PCM, 3 different statistical models of increasing complexity were constructed to adjust for potential confounding. Model 1 employed a proportional-hazards Cox regression model, with α1-adrenergic receptor antagonist use as the time-dependent exposure and time-fixed confounders.

To prevent biased estimates of association resulting from time-dependent confounders acting as mediators in the association between α1-adrenergic receptor antagonists and prostate cancer, all-cause mortality, or PCM, we additionally fit 2 marginal structural Cox models through inverse probability weighting with different degree of adjustment for confounding (models 2 and 3, respectively). Stabilized weights truncated at the first and 99th percentiles were used to mitigate the influence of extreme weights and increase the precision of the estimates (20) (Supplementary Table 1, available online). The model to estimate the denominator and the numerator of the stabilized weights was a Cox regression for the probability of being an α1-adrenergic receptor antagonist user. For model 2, the model for the denominator of the weights included the time-fixed confounders and the time-dependent exposure to 5-α reductase inhibitor as covariates. For model 3, the model for the denominator included the time-fixed confounders plus the following time-dependent confounders: exposure to a 5-α reductase inhibitor, log(PSA), and the cumulative number of PSA tests as covariates. For both models 2 and 3, the model for the numerator of the weights included only the time-fixed confounders as covariates. Finally, we fit the 2 marginal structural Cox models using the stabilized weights and a robust variance estimator. To adjust for possible confounding resulting from the use of stabilized weights, we included in the marginal structural Cox model the time-fixed confounders as covariates (20). In a sensitivity analysis to analyze the association between exposure and outcome over time, we divided follow-up time into 2-year periods by adding an interaction term connecting time period and exposure status to our models.

We modeled cause-specific hazards, which represent the instantaneous rates of the event of interest in scenarios where individuals may experience other, competing events. Cause-specific hazard ratios (HRs) and corresponding 95% confidence intervals (CIs) were reported as measures of association for all outcomes. To estimate cause-specific hazard ratios, patients experiencing a competing event were censored at the time of the competing event to remove them from the at-risk set. Schoenfeld residuals were inspected to confirm adherence to the assumption of proportional hazards. Imputation with chained equations addressed missing data for education and civil status covariates. Ten datasets were generated, and the cause-specific hazard ratios were pooled using the Rubin rule. In all analyses, 95% confidence intervals were 2 sided. All statistical analyses were conducted using R, version 4.0.4 (R Foundation for Statistical Computing, Vienna, Austria) and the *ipw,* version 1.2.1, R package (21).

## Results

The final cohort comprised 351 297 men, including 39 856 (11.3%) users of α1-adrenergic receptor antagonists (Figure 1). Median (interquartile range) follow-up for prostate cancer incidence was 8.9 (5.1-10.9) years and for all-cause mortality and PCM was 8.9 (5.2-10.9) years. The median (interquartile range) exposure time to α1-adrenergic receptor antagonists was 4.1 (1.8-7.2) years for prostate cancer incidence and 4.4 (2.0-7.6) years for all-cause and PCM.

The α1-adrenergic receptor antagonist users were generally older, had higher PSA levels, were more likely to have had a previous negative biopsy, and had a higher Charlson Comorbidity Index (Table 1). Compared with nonusers, α1-adrenergic receptor antagonist users were more likely to be prescribed 5-α reductase inhibitors, with a prevalence of 38% among α1-adrenergic receptor antagonist users compared with 3% among nonusers. Most α1-adrenergic receptor antagonist users were taking alfuzosin (95.9%), with fewer men using terazosin (3.1%) and tamsulosin (1%). Similarly, most men were prescribed finasteride (91%), whereas fewer took dutasteride (9%).

**Table 1.** Patient characteristics at inclusion

	α1-adrenergic receptor antagonist use at study inclusion	
	No	Yes
	n = 311 441	n = 39 856
Age, median (IQR), y^a^	56 (50–64)	63 (57–69)
Prostate-specific antigen level, median (IQR), ng/mL	0.98 (0.60-1.70)	1.70 (0.92-3.30)
Previous negative biopsy, No. (%)	2461 (0.8)	1047 (2.6)
Prostate cancer in a first-degree relative, No. (%)	32 024 (10)	3292 (8.3)
Highest level of education achieved, No. (%)		
Elementary school	50 360 (17)	7500 (21)
High school	125 539 (43)	15 025 (42)
University/college	118 447 (40)	13 551 (38)
Unknown	17 095	3780
Civil status, No. (%)		
Single	122 852 (40)	13 738 (35)
Partner	185 966 (60)	25 759 (65)
Unknown	2623	359
Charlson Comorbidity Index, No. (%)		
0	276 235 (89)	33 763 (85)
1	28 591 (9.2)	4965 (12)
≥2	6615 (2.1)	1128 (2.8)
Year of study entry, No. (%)		
2008-2011	174 693 (56)	30 987 (78)
2012-2015	81 449 (26)	6295 (16)
2016-2019	55 299 (18)	2574 (6.5)
5-α reductase inhibitor use, No. (%)	9604 (3.1)	15 202 (38)

a IQR = Interquartile range.

During the follow-up period, 17 356 men were diagnosed with prostate cancer. The crude incidence rate of prostate cancer was higher for men treated with α1-adrenergic receptor antagonists (133 per 10 000 person-years for users and 64 per 10 000 person-years for nonusers). The men exposed to α1-adrenergic receptor antagonists had higher International Society of Urological Pathology (ISUP) grades and T stages at diagnosis (Table 2).

**Table 2.** Patient and tumor characteristics at prostate cancer diagnosis

	α1-adrenergic receptor antagonist use at diagnosis	
	No	Yes
	n = 14 885	n = 2471
Age, median (IQR), y^a^	67 (62-73)	71 (66-76)
Prostate-specific antigen, median (IQR), ng/mL	6 (4-9)	6 (4-10)
Unknown	3157	577
Prostate volume, median (IQR), mL	37 (29-50)	43 (32-60)
Unknown	4847	900
Prostate-specific antigen density, median (IQR), ng/mL/mL	0.16 (0.11-0.25)	0.15 (0.10-0.25)
Unknown	4958	963
International Society of Urological Pathology grade, No. (%)		
1	4631 (44)	721 (43)
2	3229 (31)	478 (28)
3	1297 (12)	218 (13)
4	651 (6.2)	101 (6.0)
5	694 (6.6)	162 (9.6)
Unknown	4383	791
Clinical T stage, No. (%)		
1	9196 (66)	1497 (66)
2	3768 (27)	557 (25)
3	946 (6.8)	194 (8.5)
4	99 (0.7)	24 (1.1)
Unknown	876	199
Clinical N stage, No. (%)		
0	3418 (95)	580 (96)
1	170 (4.7)	24 (4.0)
Unknown	11 297	1867
Clinical M stage, No. (%)		
0	12 244 (97)	2164 (97)
1	400 (3.2)	75 (3.3)
Unknown	2241	232

a IQR = Interquartile range.

No evidence of an association was found between α1-adrenergic receptor antagonist use and detection of PCM, all-cause mortality, or ISUP grade 2 and higher and 3 and higher prostate cancer when employing any of the statistical models to account for confounding (Table 3). α1-Adrenergic receptor antagonist use was associated with an increased risk of ISUP grade 1 prostate cancer (HR = 1.13, 95% CI = 1.04 to 1.23) when adjusting for baseline confounders (model 1) (Table 3). When applying the marginal structural Cox model with inverse probability weighting after adjusting for baseline confounders and time-dependent 5-α reductase inhibitor use, α1-adrenergic receptor antagonist use was associated with an increased risk of prostate cancer (HR = 1.11, 95% CI = 1.06 to 1.17) and ISUP grade 1 prostate cancer (HR = 1.25, 95% CI = 1.14 to 1.36; model 2) (Table 3). The same trend continued when using the marginal structural Cox model with inverse probability weighting and adjusting for baseline confounders, time-dependent 5-α reductase inhibitor use, and PSA result (prostate cancer: HR = 1.11, 95% CI = 1.06 to 1.17; ISUP grade 1 prostate cancer: HR = 1.22, 95% CI = 1.11 to 1.33; model 3) (Table 3). In the sensitivity analysis split by follow-up time, the hazard ratios increased for all prostate cancer outcomes in all models for the first year of follow-up but were close to 1 after longer follow-up. The hazard ratios for PCM and all-cause mortality did not show a time-dependent association (Supplementary Table 2, available online).

**Table 3.** Hazard ratios for the association between α1-adrenergic receptor antagonist exposure and prostate cancer, all-cause mortality, and prostate cancer–specific mortality

	Person-years	No. of cases	Model 1^a^ HR (95% CI)	Model 2^b^ HR (95% CI)	Model 3^c^ HR (95% CI)
All prostate cancer					
No use	2 324 216	14 885	1.00 (Referent)	1.00 (Referent)	1.00 (Referent)
α1-adrenergic receptor antagonist use	185 765	2471	1.03 (0.98 to 1.07)	1.11 (1.06 to 1.17)	1.11 (1.06 to 1.17)
International Society of Urological Pathology grade 1					
No use	2 324 216	4631	1.00 (Referent)	1.00 (Referent)	1.00 (Referent)
α1-adrenergic receptor antagonist use	185 765	721	1.13 (1.04 to 1.23)	1.25 (1.14 to 1.36)	1.22 (1.11 to 1.33)
International Society of Urological Pathology grade ≥2					
No use	2 324 216	5871	1.00 (Referent)	1.00 (Referent)	1.00 (Referent)
α1-adrenergic receptor antagonist use	185 765	959	0.93 (0.87 to 1.00)	1.01 (0.94 to 1.09)	0.93 (0.86 to 1.00)
International Society of Urological Pathology grade ≥3					
No use	2 324 216	2642	1.00 (Referent)	1.00 (Referent)	1.00 (Referent)
α1-adrenergic receptor antagonist use	185 765	481	0.98 (0.89 to 1.08)	1.03 (0.92 to 1.15)	0.91 (0.82 to 1.02)
All-cause mortality					
No use	2 395 578	31 899	1.00 (Referent)	1.00 (Referent)	1.00 (Referent)
α1-adrenergic receptor antagonist use	194 951	5188	1.01 (0.98 to 1.05)	1.01 (0.97 to 1.04)	1.02 (0.99 to 1.06)
Prostate cancer mortality					
No use	2 395 578	642	1.00 (Referent)	1.00 (Referent)	1.00 (Referent)
α1-adreneric receptor antagonist use	194 951	163	1.00 (0.84 to 1.18)	1.08 (0.90 to 1.30)	0.90 (0.74 to 1.09)

a Cox proportional hazards model—adjusted for time-fixed baseline covariates (age, prostate-specific antigen level, previous negative biopsy result, prostate cancer heredity, educational attainment, civil status, Charlson Comorbidity Index, year of study entry. CI = confidence interval; HR = hazard ratio.

b Marginal structural model created with inverse probability weighting to adjust for baseline covariates and time-dependent 5-α reductase inhibitor exposure.

c Marginal structural model created with inverse probability weighting to adjust for baseline covariates and time-dependent 5-α reductase inhibitor exposure, prostate-specific antigen level, and cumulative number of prostate-specific antigen tests.

**Figure 2.**
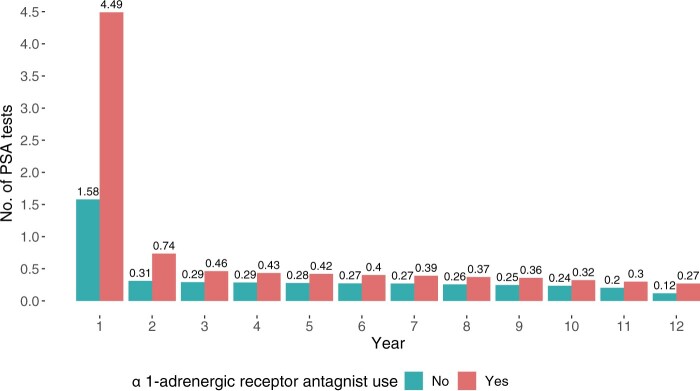
Mean number of PSA tests per year for users of α1 blockers and nonusers (defined by accumulated person-time as exposed and unexposed). PSA = prostate-specific antigen.

Throughout the follow-up, men treated with α1-adrenergic receptor antagonists consistently underwent more PSA tests. The most significant difference in mean PSA tests per year was observed during the first year of treatment or nontreatment, with α1-adrenergic receptor antagonist users averaging 4.49 PSA tests per year compared with 1.58 PSA tests per year among nonusers (Figure 2).

## Discussion

This study represents the largest population-based study conducted thus far on the relationship between α1-adrenergic receptor antagonists and prostate cancer mortality. Although early in vitro and in vivo studies showed promise in potentially using α1-adrenergic receptor antagonists as chemopreventive agents, our results fail to support this hypothesis; rather, they indicate that there is no significant change in prostate cancer mortality between treated and untreated men. Nevertheless, it is worth noting that we observed a slight increase in the incidence of indolent prostate cancers among α1-adrenergic receptor antagonist users, even after adjusting for the frequency of PSA testing. This finding may be attributed to more clinical visits to urologists for α1-adrenergic receptor antagonist users, increasing the likelihood of undergoing prostate biopsies. Considering the widespread use of the medication for managing lower urinary tract symptoms, our findings have significant clinical implications, confirming that α1-adrenergic receptor antagonists cannot serve as a repurposed strategy for the chemoprevention of prostate cancer.

Early clinical trials had demonstrated that doxazosin and terazosin, both quinazoline α1-adrenergic receptor antagonists, exhibited in vitro antitumor activity against prostate cancer cells through an α1-adrenergic receptor antagonist–independent mechanism (22,23). In contrast, tamsulosin, which belongs to the sulphonamide class of α antagonists, did not show the same antitumoral effect (24).

The quinazoline-mediated apoptosis is believed to occur because of the upregulation of transforming growth factor-β1, a potent regulator of prostate cell proliferation and apoptosis. transforming growth factor-β1 serves as a potent regulator of prostate proliferation and apoptosis, given its ability to inhibit cell proliferation and induce apoptosis (25). An immunohistochemical analysis of human prostate specimens affected by BPH indicated that treatment with terazosin led to a significant reduction in microvessel density within the prostate tissue. This effect is mediated by the downregulation of vascular endothelial growth factor (26,27). In prostate cancer, vascular endothelial growth factor expression correlates with tumor stage, grade, microvessel density, and clinical outcome (28). This finding prompted early investigations into the potential of quinazoline-based α1-adrenergic receptor antagonists for prostate cancer chemoprevention, given their ability to block various mechanisms involved in prostate cancer growth and proliferation. These medications have also shown promise in reducing the risk of bladder cancer through their antiangiogenic mechanisms (29).

This observational study is differentiated from previous epidemiological studies on α1-adrenergic receptors on multiple points. First, preclinical studies had indicated a potential antitumoral effect primarily for quinazoline-based α1-adrenergic receptor antagonists. We were able to test this hypothesis because most α1-adrenergic receptor antagonist users in our study were taking quinazoline-based drugs. In a case-control study by Murtola et al. (12), where more than 90% of men were exposed to tamsulosin, an increased risk of prostate cancer was found among regular users (odds ratio = 1.79, 95% CI = 1.67 to 1.91). In contrast, a later cohort study also by Murtola et al.—the European Randomized Study of Screening for Prostate Cancer screening cohort—found a statistically significant decrease in clinically significant prostate cancer. Once again, it is important to note that the majority of α1-adrenergic receptor antagonist users were taking tamsulosin (13). In addition, we were able to adjust for the use of 5-ɑ reductase inhibitor, previously shown to decrease detection of low-grade prostate cancer and potentially PCM (30-34). In a US Department of Veterans Affairs cohort study, Harris et al. (10) reported a risk ratio for quinazoline-based α1-adrenergic receptor antagonist users of 0.69 (95% CI = 0.53 to 0.88). That study, however, was limited by its unadjusted design, where men may have also been exposed to 5-ɑ reductase inhibitors. In a well-designed population-based matched case-control study conducted by Robinson et al., an increased risk of prostate cancer was found in men exposed to α1-adrenergic receptor antagonists (odds ratio = 1.33, 95% CI = 1.27 to 1.39), statistically significant for Gleason Grade Groups 1 and 2, after adjusting for 5-ɑ reductase inhibitor use and previous biopsies. No adjustment was made for PSA levels, however, and the authors did not specify the α1-adrenergic receptor antagonists included (11). For PCM in men exposed to α1-adrenergic receptor antagonists, findings have been inconsistent. A prospective cohort study by Preston et al. found no difference in either prostate cancer incidence or PCM, adjusting for multiple socioeconomic factors, 5-ɑ reductase inhibitor use, and diagnostic intensity with PSA and biopsies. A limitation of that study, however, is its reliance on health questionnaires for its confounders (14). Other studies, each with its limitations, have found increased (35) and decreased (36,37) PCM in men exposed to α1-adrenergic receptor antagonists. What distinguishes our study from those prior investigations is access to diagnostic intensity data, including PSA levels and biopsies, as well as the use of a drug registry with near-complete coverage.

It is essential to highlight the role of PSA testing, which both increases the probability of prostate cancer diagnosis and reduces the risk of PCM (38,39). Therefore, the influence of PSA and testing intensity in this study was crucial. Our study revealed differences in PSA testing intensity between exposed and unexposed men, with the largest difference in the first year of drug use. The increased testing in the first year may be attributed to the typical lower urinary tract symptoms these individuals experience, leading health-care professionals to prescribe the drug and recommend PSA testing. In subsequent years, the increased testing frequency can be attributed to various factors: the older age of men prescribed the drug, higher PSA levels, and more frequent monitoring by either general practitioners or urologists. Moreover, receiving a BPH diagnosis may be distressing for some men, motivating them to seek regular PSA testing more proactively. The differences in PSA testing intensity between the exposed and unexposed groups underscore the complexity of PSA screening and the significance of shared decision making.

The strengths of this study include its large study cohort, real-world setting, long-term follow-up, and registries with excellent coverage. We were able to obtain accurate data on drug use for both α1-adrenergic receptor antagonists and 5-ɑ reductase inhibitors as well as confirmation of filled prescriptions. Additionally, we had access to all PSA tests performed in the region during the study period and could adjust the analysis accordingly. We were also able to adjust our analyses for a previous negative biopsy, which is significantly associated with a lower risk of both low-grade and high-grade prostate cancer (40). Some limitations are associated with our study as well. First, even after adjusting for PSA testing, there is still a possible detection bias where patients with lower urinary tract symptoms are more likely to visit a urologist and undergo a prostate biopsy. In addition, the sensitivity analysis, split by follow-up time, showed slight increases in hazard ratios for the first 2 years for prostate cancer for all models, even when adjusting for 5-ɑ reductase inhibitor exposure and PSA testing intensity, likely due to residual detection bias. Second, although choosing an intention-to-treat study design reduces healthy user and sick stopper bias, it also increases misclassification, potentially biasing our results toward the null (41). Third, despite the long follow-up in the present study, considering that prostate cancer is predominantly a slow-growing malignancy, a longer follow-up would ideally be evaluated to see an impact from the long-term exposure to α1-adrenergic receptor antagonists. Further, because the Swedish Prescribed Drug Registry was initiated in July 2005, participants could have been exposed to α1-adrenergic receptor antagonists before this. In addition, we were unable to adjust for confounders such as body mass index, smoking status, and physical activity. Finally, modeling cause-specific hazards does not rely on the assumption of independent competing events—an assumption that cannot be empirically tested and likely did not hold in our study. Only under this assumption can the cause-specific hazards be interpreted because the rates would have been observed in the hypothetical situation, where only the event of interest occurs, without the influence of other competing events (42).

The results of this large population-based cohort study suggest that there is no link between the use of α1-adrenergic receptor antagonists and prostate cancer mortality. Given the widespread use of α1-adrenergic receptor antagonists for the treatment of lower urinary tract symptoms/BPH, our findings support the notion that these medications are unlikely to serve as potential chemopreventive agents or treatment strategies in the medical management of prostate cancer. Nonetheless, we encourage further preclinical research to explore the potential antitumoral effects of α1-adrenergic receptor antagonists. Importantly, we did not find evidence that contradicts the hypothesis that this medication is safe in terms of prostate cancer and overall mortality.

## Supplementary Material

djae108_Supplementary_Data

## Data Availability

Access to the data supporting this study is available through the included Swedish registers. Ethical and legal constraints may affect data availability, however, and specific ethical permissions for the study are necessary.
