# Supplementary material for: Prostate cancer incidence and mortality in men exposed to α1-adrenergic receptor antagonists
Source: J Natl Cancer Inst. 2024 May 8;116(9):1459–65. doi: 10.1093/jnci/djae108 (PMC11378311; doi:10.1093/jnci/djae108)
Supplement: djae108_Supplementary_Data [file djae108_supplementary_data.docx]

**Supplementary material**

Prostate Cancer Incidence and Mortality in Men Exposed to α1-Adrenoceptor Antagonists

Lars Björnebo MD, MSc^1^, Shirin Razdan MD^2^, Andrea Discacciati PhD^1^, Thorgerdur Palsdottir PhD^1^, Markus Aly MD PhD^3^, Tobias Nordström MD PhD^1^, Martin Eklund PhD^1^, Dara Lundon MD PhD^2^, Henrik Grönberg MD PhD^1^, Ash Tewari MD^2,4^, Peter Wiklund MD PhD^2^, Natasha Kyprianou MD PhD^2,4^ *, Anna Lantz MD PhD^1,3^*

*Both authors contributed equally as corresponding authors

*^1^ Department of Medical Epidemiology and Biostatistics, Karolinska Institutet, Stockholm, Sweden*

*^2^ Department of Urology, Icahn School of Medicine at Mount Sinai, New York, USA*

*^3^ Department of Urology, Karolinska University Hospital Solna, Sweden*

*^4^ Tisch Cancer Institute, Icahn School of Medicine at Mount Sinai, New York, USA*

**Supplementary Table 1 – Estimated weights^*^ for the marginal structural models with inverse probability weighting.**

|  | Model 2^a^ (incidence) | Model 2^a^ (mortality) | Model 3^b^ (incidence) | Model 3^b^ (mortality) |
| --- | --- | --- | --- | --- |
| Maximum | 1.41 | 1.42 | 1.74 | 1.66 |
| 75th percentile | 1.00 | 1.00 | 1.01 | 1.00 |
| Median | 1.00 | 1.00 | 1.00 | 1.00 |
| 25th percentile | 0.99 | 0.99 | 0.98 | 0.98 |
| Minimum | 0.29 | 0.28 | 0.26 | 0.25 |

^*^The reported weights are stabilized and truncated at the 1st and 99th percentiles

^a^Marginal structural model created with inverse probability weighting (IPW) to adjust for baseline covariates and time-dependent 5-ARI exposure

^b^Marginal structural model created with IPW to adjust for baseline covariates and time-dependent 5-ARI exposure, PSA, and cumulative number of PSA tests

**Supplementary Table 2 - Hazard ratios split by follow-up time for the association between α1-antagonist exposure and prostate cancer, all-cause mortality, and prostate cancer mortality; ISUP = International Society of Urological Pathology, HR = hazard ratio, CI = confidence interval**

|  | Model 1^a^  HR (95% CI) | Model 2^b^  HR (95% CI) | Model 3^c^  HR (95% CI) |
| --- | --- | --- | --- |
| All prostate cancer |  |  |  |
| No use | 1.00 (ref) | 1.00 (ref) | 1.00 (ref) |
| α1-antagonist use (0-2 yrs)^d^ | 1.39 (1.25-1.56) | 1.55 (1.38-1.75) | 1.56 (1.38-1.76) |
| α1-antagonist use (2-4 yrs)^d^ | 1.03 (0.93-1.15) | 1.15 (1.03-1.28) | 1.13 (1.01-1.27) |
| α1-antagonist use (4-6 yrs)^d^ | 0.98 (0.89-1.08) | 1.06 (0.96-1.18) | 1.07 (0.96-1.18) |
| α1-antagonist use (6-8 yrs)^d^ | 0.92 (0.84-1.02) | 0.98 (0.88-1.08) | 1.02 (0.91-1.13) |
| α1-antagonist use (8-10 yrs)^d^ | 1.04 (0.94-1.16) | 1.11 (0.99-1.24) | 1.16 (1.03-1.29) |
| α1-antagonist use (10-12 yrs)^d^ | 0.92 (0.80-1.06) | 0.98 (0.85-1.14) | 0.91 (0.78-1.06) |
| ISUP 1 |  |  |  |
| No use | 1.00 (ref) | 1.00 (ref) | 1.00 (ref) |
| α1-antagonist use (0-2 yrs)^d^ | 1.64 (1.37-1.95) | 1.85 (1.54-2.23) | 2.00 (1.64-2.42) |
| α1-antagonist use (2-4 yrs)^d^ | 1.01 (0.84-1.22) | 1.15 (0.94-1.40) | 1.13 (0.92-1.38) |
| α1-antagonist use (4-6 yrs)^d^ | 0.92 (0.78-1.10) | 0.98 (0.82-1.18) | 0.95 (0.79-1.15) |
| α1-antagonist use (6-8 yrs)^d^ | 1.00 (0.84-1.20) | 1.08 (0.89-1.31) | 1.06 (0.87-1.29) |
| α1-antagonist use (8-10 yrs)^d^ | 1.36 (1.10-1.69) | 1.50 (1.20-1.87) | 1.45 (1.15-1.83) |
| α1-antagonist use (10-12 yrs)^d^ | 1.17 (0.87-1.57) | 1.34 (0.98-1.81) | 1.04 (0.75-1.42) |
| ISUP 2 |  |  |  |
| No use | 1.00 (ref) | 1.00 (ref) | 1.00 (ref) |
| α1-antagonist use (0-2 yrs)^d^ | 1.26 (1.04-1.51) | 1.38 (1.13-1.68) | 1.25 (1.03-1.54) |
| α1-antagonist use (2-4 yrs)^d^ | 1.02 (0.87-1.21) | 1.17 (0.99-1.39) | 1.10 (0.92-1.31) |
| α1-antagonist use (4-6 yrs)^d^ | 0.92 (0.79-1.07) | 1.01 (0.86-1.19) | 0.97 (0.82-1.14) |
| α1-antagonist use (6-8 yrs)^d^ | 0.77 (0.66-0.91) | 0.80 (0.67-0.95) | 0.78 (0.66-0.93) |
| α1-antagonist use (8-10 yrs)^d^ | 0.89 (0.75-1.05) | 0.93 (0.78-1.11) | 0.84 (0.70-1.00) |
| α1-antagonist use (10-12 yrs)^d^ | 0.90 (0.74-1.10) | 0.98 (0.80-1.21) | 0.82 (0.66-1.02) |
| ISUP 3+ |  |  |  |
| No use | 1.00 (ref) | 1.00 (ref) | 1.00 (ref) |
| α1-antagonist use (0-2 yrs)^d^ | 1.35 (1.04-1.76) | 1.42 (1.07-1.89) | 1.23 (0.92-1.63) |
| α1-antagonist use (2-4 yrs)^d^ | 1.12 (0.89-1.41) | 1.27 (0.99-1.61) | 1.10 (0.86-1.40) |
| α1-antagonist use (4-6 yrs)^d^ | 0.89 (0.71-1.11) | 0.97 (0.77-1.23) | 0.92 (0.72-1.18) |
| α1-antagonist use (6-8 yrs)^d^ | 0.72 (0.57-0.91) | 0.74 (0.57-0.95) | 0.71 (0.55-0.92) |
| α1-antagonist use (8-10 yrs)^d^ | 1.04 (0.83-1.31) | 1.04 (0.81-1.32) | 0.91 (0.71-1.17) |
| α1-antagonist use (10-12 yrs)^d^ | 1.01 (0.76-1.34) | 1.00 (0.74-1.35) | 0.83 (0.61-1.13) |

| All-cause mortality |  |  |  |
| --- | --- | --- | --- |
| No use | 1.00 (ref) | 1.00 (ref) | 1.00 (ref) |
| α1-antagonist use (0-2 yrs)^d^ | 0.95 (0.87-1.04) | 0.93 (0.85-1.03) | 0.96 (0.87-1.06) |
| α1-antagonist use (2-4 yrs)^d^ | 0.99 (0.92-1.07) | 1.00 (0.92-1.08) | 1.02 (0.94-1.11) |
| α1-antagonist use (4-6 yrs)^d^ | 1.04 (0.98-1.12) | 1.04 (0.96-1.11) | 1.04 (0.96-1.12) |
| α1-antagonist use (6-8 yrs)^d^ | 1.00 (0.93-1.06) | 0.99 (0.92-1.06) | 1.01 (0.94-1.08) |
| α1-antagonist use (8-10 yrs)^d^ | 1.06 (0.99-1.13) | 1.05 (0.98-1.13) | 1.06 (0.99-1.14) |
| α1-antagonist use (10-12 yrs)^d^ | 1.03 (0.95-1.13) | 1.02 (0.93-1.11) | 1.06 (0.97-1.17) |
| Prostate cancer mortality |  |  |  |
| No use | 1.00 (ref) | 1.00 (ref) | 1.00 (ref) |
| α1-antagonist use (0-2 yrs)^d^ | 1.13 (0.48-2.63) | 1.31 (0.53-3.24) | 1.06 (0.41-2.78) |
| α1-antagonist use (2-4 yrs)^d^ | 1.10 (0.67-1.79) | 1.22 (0.73-2.05) | 0.92 (0.53-1.58) |
| α1-antagonist use (4-6 yrs)^d^ | 1.50 (1.06-2.12) | 1.57 (1.08-2.28) | 1.35 (0.92-1.98) |
| α1-antagonist use (6-8 yrs)^d^ | 0.76 (0.52-1.11) | 0.87 (0.59-1.30) | 0.76 (0.51-1.15) |
| α1-antagonist use (8-10 yrs)^d^ | 0.90 (0.61-1.32) | 0.91 (0.60-1.38) | 0.78 (0.51-1.19) |
| α1-antagonist use (10-12 yrs)^d^ | 0.88 (0.58-1.33) | 0.98 (0.63-1.52) | 0.78 (0.49-1.23) |
| ^a^Cox proportional hazards model – adjusted for time-fixed baseline covariates (age, PSA, previous negative biopsy, prostate cancer heredity, education, civil status, Charlson Comorbidity Index, year of study entry  ^b^Marginal structural model created with inverse probability weighting (IPW) to adjust for baseline covariates and time-dependent 5-ARI exposure  ^c^Marginal structural model created with IPW to adjust for baseline covariates and time-dependent 5-ARI exposure, PSA, and cumulative number of PSA tests  ^d^Stratified by follow-up time (0-2 yrs, 2.-4 yrs, 4-6 yrs, 8-10 yrs, and 10-12 yrs) | | | |
